# Matrix-matched quantification of volatile organic compounds (VOCs) in gluten free flours and bakery products

**DOI:** 10.1016/j.fochx.2024.101399

**Published:** 2024-05-21

**Authors:** Antonella Porrello, Santino Orecchio, Antonella Maggio

**Affiliations:** Department of Biological, Chemical and Pharmaceutical Sciences and Technologies (STEBICEF), University of Palermo, Viale delle Scienze, Palermo, building 17, Italy

**Keywords:** Food analysis, Gluten free, Analytical method, VOCs, HS-SPME/GC–MS

## Abstract

The aim of this study deals with characterize the volatile profiles of gluten free flours and bakery products. An appropriate HS-SPME/GC–MS methods for the quantification analyses was performed and corn starch solid as standards was used. 34 different samples were analysed, and 127 compounds distributed in 4 classes (alcohols, aldehydes and ketones, heterocyclic compounds, and terpenes), that make up the aroma of these gluten free, were identified. The developed method is characterized by detection limits of 0.0004 and 0.0047 mg/kg for camphor and pyrazine, respectively, and linearity of quantification standards were between 0.990 and 0.998 for a range of 3–50 mg/kg.

## Highlights

• Matrix-matched quantification of volatile organic compounds of gluten free bakery products
• 127 compounds divided into 9 classes were identify.
• Headspace solid-phase microextraction coupled to GC–MS has been used.

## 1 Introduction

Flavor compounds was investigated due the principal characteristic of their interaction with the human olfactory system. The concept of flavor is thought to be a sensation caused by the ingestion of food in the mouth and is perceived mainly by the sense of taste and smell, so most of the flavor research has so far focused on aroma compounds (odorants). Volatile organic compounds (VOCs) contribute greatly to its acceptance by consumers. The volatile organic compounds that occur in food can be originated from raw materials or are formed during fermentation, lipid oxidation, enzymatic or thermal reactions, for example Maillard reactions (Drakula et al., 2021). The growth of knowledge in the aroma of foods represents a useful tool for its improvement, so it's a topic of growing interest in scientific research (Pacynski, Zawirska Wojtasiak, & Mildner-Szkudlarz, 2015) and in the food industries. The VOCs profile varies according to raw material geographical origin and are important (De Flaviis, Sacchetti, & Mastrocola, 2021) to obtain information on food traceability. The volatile profile of a food is also determined by transformation processes to which it is subjected (Cirlini, & Dall'Asta, C., Silvannini, A., Beghè, D., Fabbri, A., Galaverna, G., & Ganino, T., 2012) and therefore information about processing steps (Pico, Martinez, Bernal, & Gomez, 2017) is possible to obtain.

Since the begin of XXI century, >60 reviews have been published on food flavors. Most of them dealt with the flavor of certain foods (e.g. wine, cheese, yogurt, butter, fruit (apple, strawberry, kiwi)), olive oil, beer, grape juice, hand-squeezed orange juice, cocoa, chocolate, tea, bread, rice, herbs, and compounds responsible for the formation of off-flavors in various foods.

Gluten-free foods are still poorly investigated group in terms of their volatile profile. Nowadays, celiac disease is widespread worldwide affecting about 1% of the world's population (Makharia et al., 2022). The increase in the number of diagnoses of celiac disease has consequently contributed to the production of numerous commercially available gluten-free food products. The production of gluten-free products is mainly based on rice and corn and to a lesser extent on other naturally gluten-free cereals which typically don't offer the same performance of wheat as the production of bakery products. Therefore, these products contain a high content of fat, sugar, and salt to improve the organoleptic characteristics (Nissen, Bordoni, & Gianotti, 2020).

To obtain information on the quality of gluten-free foods, several studies have been conducted highlighting the composition in macro- and micro-nutrients (Orecchio et al., 2014; Orecchio et al., 2015) and fatty acids (Maggio & Orecchio, 2018) of these foods, so, in addition to these, the study of volatile organic compounds (VOCs) represents a significant new aspect on which to base a study. For gluten-free products the trend is to increase the aroma, favouring the production of specific VOCs through the addition of precursors. This can lead the development of undesirable compounds, the so-called off flavors, which can for example derive from other processes. The identification of off flavors is important for designing aroma-enhancing strategies (Rajhi, Baccouri, Rajhi, Mhadhbi, & Flamini, 2021).

In the last decade, solid phase micro extraction (SPME) combined with GC/MS has been frequently used for volatile compounds analyses because it is simple, highly sensitive, solvent less technique and with possibility of automation (Jelen, Majcher, & Dziadas, 2012). However, there are not many studies that take the matrix effect into account when developing a properly quantification.

In this work, particular attention was paid to the development of an appropriate quantification method to the development standards that simulate the matrix effect.

Specifically, the following study was aimed to use a novel analytical method to characterize the profiles of volatile components of gluten-free foods to evaluate the potential of fingerprinting to differentiate foods of similar nature and also to highlight the possible presence of compounds responsible for organoleptic alterations, precursors of flavors, contaminants and key odours.

## 2 Material and methods

### 2.1 Samples

In this work, 8 samples of flour (4 gluten-free and 4 traditional), 22 bakery products (cookies, crackers, etc.) and 4 ingredients were considered (Table 1). The samples were purchased directly from pharmacies and supermarkets in the province of Palermo (Italy).

**Table 1:** List of samples analysed, and their respective identification names used in the text.

	Sample	Identification name (*)	Gluten (**)
**1**	Wheat flour 00	F00_CO	P
**2**	Wheat flour 0	F0_CO	P
**3**	Wheat flour Manitoba 0	FM0_CO	P
**4**	Wheat flour Manitoba 0	FM0_CA	P
**5**	Rice flour	FR_CO	A
**6**	Rice flour	FR_SA	A
**7**	Rice flour	FR_DE	A
**8**	Buckwheat flour	FGS_SA	A
**9**	Bread mix	PREP_pane_SA	A
**10**	Bread mix	PREP_pane_SH	A
**11**	Pastry mix	PREP_dolci_CO	A
**12**	Flour mix	MIX_F_SH	A
**13**	White bread	PB_NU	A
**14**	White bread	PB_CE	A
**15**	Wholemeal bread	PI_CO	A
**16**	Chia seeds bread	PS_SH	A
**17**	Rice and corn pasta	PASTA_SE	A
**18**	Rice and corn crackers	GALLETTE_SC	A
**19**	Breadcrumb	PG_SE	A
**20**	Breadcrumb	PG_NU	A
**21**	Rosemary crackers	CR_CE	A
**22**	Rosemary crackers	CR_NU	A
**23**	Rosemary breadsticks	GR_SC	A
**24**	Spicy crackers	CP_DO	A
**25**	Shortbread	B_GU	A
**26**	Cookies with chocolate chips	BG_GU	A
**27**	Cookies with chocolate chips	BG_CO	A
**28**	Plumcake	PLUM_SO	A
**29**	Shortbread with chocolate chips	FG_GA	A
**30**	Cocoa shortbread with chocolate chips	FCG_CO	A
**31**	Bitter cocoa	Cocoa	A
**32**	Dried rosemary	Rosemary	A
**33**	Sweet paprika powder	Paprika	A
**34**	Vanillin	Vanillin	A

(*) the suffix of the identifying name indicates the brand; (**) P = present, A = absent.

### 2.2 Sample preparation

The volatile organic compounds were extracted with solid-phase micro extraction and analysed with gas chromatography/mass spectrometry (SPME-GC/MS). For each analysis, 2.0 g of sample, representative of the sample, was placed in a 20 mL SPME glass vial (Gerstel, 75.5 × 22.5 mm) previously placed in an oven at 100 °C for at least 12 h. Pretreatment wasn't necessary for the flour products. The bakery products (cookies, crackers, etc.) were properly ground and homogenized with porcelain mortar, except for the bread samples for which the grinding was done with the aid of a small coffee grinder. All analyses were carried out in triplicate. To avoid contamination, all necessary laboratory procedures were put in place. To control heating of the samples, the temperature was monitored.

### 2.3 Headspace solid-phase micro extraction (HS-SPME)

The headspace spontaneous volatile emission of the flours and crushing snacks were sampled by means of HS-SPME. Triplicates were assessed for each sample. For all analyses, an automated SPME holder (Supelco®, Bellefonte, PA, USA) and a 50/30 μm divinylbenzene (DVB)/carbowax (CAR)/polydimethylsiloxane (PDMS) fiber of 1 cm length was used. For extraction, each sample (2 g) was placed in a 20 mL vial (Gerstel,75.5 × 22.5 mm). The samples were equilibrated at 60 °C for 10 min. The SPME fiber was exposed to the sample for 30 min in the headspace maintained at 60 °C. From the fiber, compounds were desorbed for 10 min and transferred to the column through a splitless injector maintained at 250 °C.

### 2.4 Gas chromatography coupled with mass spectrometry (GC–MS)

The quantification of volatile compounds was performed using a gas chromatograph (Agilent 7000C) equipped with an apolar capillary column (DB-5MS) in fused silica (30 m × 0.25 mm i.d.; 0.25 μm film thickness) (Santa Clara, CA, USA) coupled to an MSD 5973 triple quadrupole detector (Mass Selective Agilent). Some of the instrumental parameters are given below:

• ionization voltage: 70 eV;
• electron multiplier energy: 2000 V;
• transfer line temperature: 270 °C;
• solvent delay: 3 min;
• helium was used as carrier gas with a flow rate of 1 mL/min.

The column temperature was initially kept constant at 40 °C for 2 min (during splitless injection), then by increasing the temperature by 4 °C/min it was set to 60 °C, at which it was kept constant for 2 min. Increasing the temperature by 2 °C/min, it was raised to 90 °C, from 190 °C to 230 °C, increasing by 5 °C/min and finally the left at 230 °C for 15 min. The analytes in the fiber were automatically injected at 250 °C with the splitless mode.

The mass spectrometer was set in MS mode to acquire all mass-to-charge ratios from 35 to 450 amu (0.1 amu). Most of the compounds in the volatile profile were identified based on their mass spectra, compared with those in the NIST Chemistry WebBook. Some analytes whose pure compounds I had were confirmed using retention times and mass spectra recorded.

### 2.5 Standards preparation

Compounds used as standards: 2-octanol (98%) CAS n.123–96-6, 2-nonanaone (99%) CAS n. 821–55-6, camphor (99%) CAS n. 464–48-2, pyrazine (99%) CAS n. 290–37-9.

For the preparation of standards ethanol and deionized water were used pure or in mixture. All the standard solution were prepared at 200 mg/L concentration. For 2-nonanone and pyrazine standards ethanol was used as solvent, while 2-octanol standard was prepared using an 8:2 mixture of ethanol and deionized water.

Matrix-matched calibration method was used for quantification. To simulate the matrix effect, among different types of solids, corn starch was chosen, which volatile compounds are negligibles by preliminary SPME-GC/MS analyses carrier out by same procedure of samples. In fact, the straight lines obtained by an external standard can only provide accurate quantification if the matrix is fully simulated. Since not all standards were available, it was chosen to use one compound for each of the main classes identified in the samples. For the calibration straight lines, the compounds listed in Fig. 3 were used and five standards were prepared for each of them at different concentrations (3.12; 6.25; 12.5; 25; 50 mg/Kg) from a 200 ppm solution (Fig. 3).

**Fig. 1:**
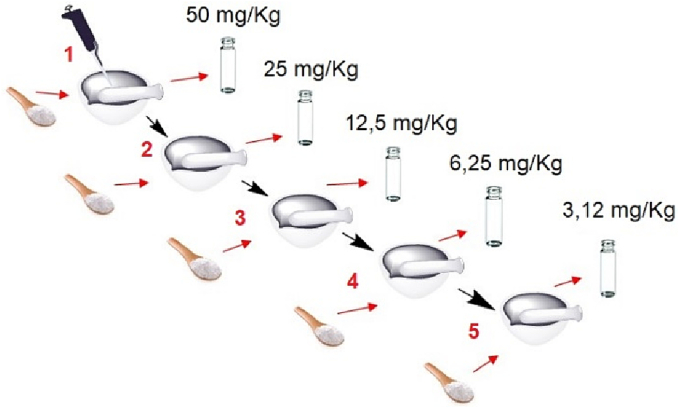
Scheme of the procedure used for the preparation of starch standards.

The procedure for preparing the 5 standards for each analyte (except camphor) is schematized in Fig. 1.

In a mortar, 1 mL of 200 ppm solution was added to 4 g of corn starch; the mixture was homogenized, resulting in a standard at a concentration of 50 mg/Kg. An aliquot (2 g) was transferred to a vial for analysis while 2 g of corn starch was added to the remainder (2 g) to obtain the standard at 25 mg/Kg. The same procedure was applied to obtain the more dilute standards. For camphor, being in a solid state, 0.2 mg of it was mixed with 4 g of corn starch, thus obtaining the 50 mg/Kg standard, after which the same procedure was followed as described above.

To avoid contamination, all necessary laboratory procedures were put in place. To control heating of the samples, the temperature was monitored.

## 3 Results and discussion

The volatile profiles of 34 samples (Table 1) of various ingredients and bakery products were characterized and 127 volatile organic compounds were identified by HS-SPME coupled with GC/MS.

To have appropriate calibration function, different calibration methodologies can be used, bearing in mind factors like the analytes nature, the availability of commercial standards and the possibility of disturbance by other components, considered them individually (interferent) or in a whole way (matrix effect) (Cuadros-Rodríguez, Bagur-González, Sánchez-Viñas, González-Casado, & Gómez-Sáez, 2007). In food analysis, consider matrix in quantification is necessary to perform a correct analysis. For this reason, matrix matched calibration it was use in this study. The matrix choice is dependent of its representativeness is usually determined among similar biological material and content in constituents like, water, acids, sugars, lipids, secondary plant metabolites, etc. In this work, to individuate the most convenient solid support different type of starch was analysed using the same procedure used for all samples. Corn starch was identified as a solid support for quantification. Although this is an approximation, the choice was made considering the similarity between starch and samples, particularly with flours and specifically corn starch, compared to the others, had the lowest number of volatile compounds, all in irrelevant amounts.

After a preliminary qualitative investigation, the four compounds used as standards were selected, each representing one of the classes identified in the samples.

The analytical method was optimized according to the nature of the samples and analytes. The extraction temperature of 60 °C allows heating of the sample to concentrate the compounds in the gas phase without inducing degradation processes that would affect the accuracy of the results.

The fiber used is a multicomponent chosen because it provides efficient extraction for a wide range of analytes with different polarity and molecular weight.

The chosen delay time does not allow to quantify analytes with retention times lower than 3 min. In this range, analysing some samples of packaged bread, were eluted ethanol, acetic acid known to be present in this type of samples and not significant for the purposes of this research.

For the evaluation of the goodness of the calibration method used, linearity, limit of detection (LOD) and limit of quantification (LOQ) were calculated on the values obtained from the analysis of the standards.

The limits of detection (LOD) and quantification (LOQ) of the method used in this study were calculated by the analysis of 8 blacks (air in the empty vial). As shown in Fig. 4, in the chromatograms of whites only the peak areas at the same RT of the 4 standards (2-octanol, 2-nonanone, camphor and pyrazine) have been considered, assuming that the same limits apply to the whole class of compounds to which each standard belongs.

Linear regression was used to evaluate the calibration method linearity. Concentration ranges were chosen between 3.12 and 50 mg/kg. The correlation coefficients of the calibration lines of the compounds used as standard are between 0.990 and 0.998, for 2-nonanone and 2-octanol respectively.

### 3.1 Alcohols

**Fig. 2:**
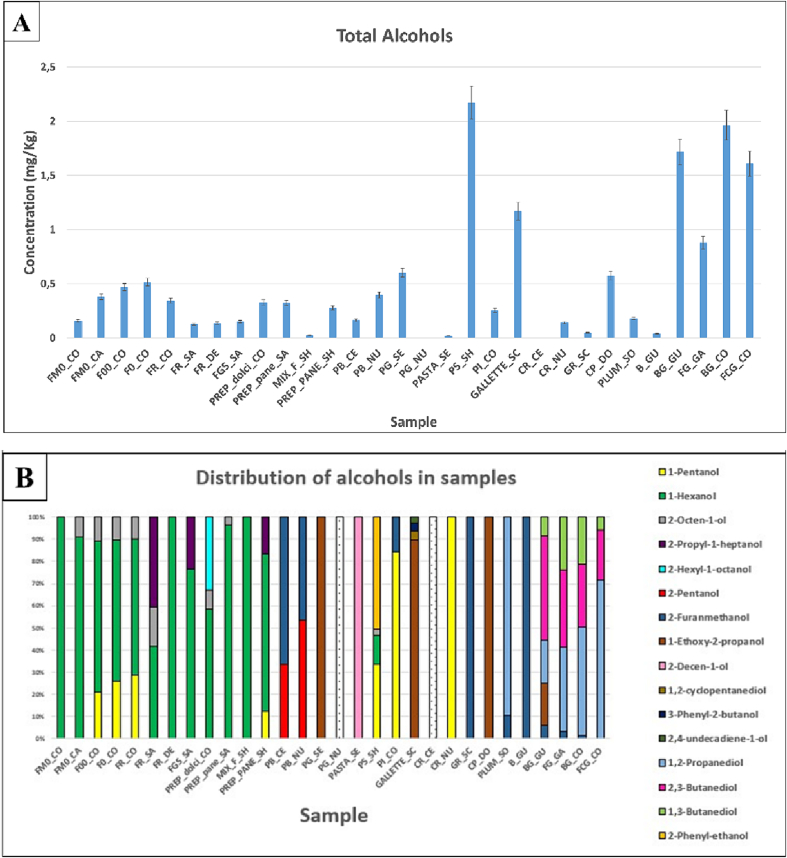
Total concentration (mg/Kg) of alcohols in the samples (A), percentage distributions of alcohols in samples (B).

Using 2-octanol calibration line, the total concentrations of the alcohols present in the samples were calculated. As can be seen from Fig. 2A, the concentrations are between the limit of quantification (PG_NU) and 2.2 mg/Kg (PS_SH).

As can be seen from Fig. 2A, alcohols, are present in almost all the samples. This result is in good agreement with what has already been pointed out by Hansen & Hansen, 1994, which identifies this class of compounds in several flours (Hansen & Hansen, 1994) in which they could have been formed to chemical oxidation processes of lipids or by microorganisms (Maire, Rega, Soto, & Giampaoli, 2013). The total alcohols concentration is generally higher in final products than in flours. This evidence could be justified considering the use of yeast in the production of final foods (bread, etc.) or even by the presence of other microorganisms. The 1-Hexanol is present and predominant alcohol in all the analysed samples, ranging from 0.026 mg/Kg (MIX_F_SH) to 0.35 mg/Kg (FM0_CA).

Four of the samples purchased from the same distributor (CO) are characterized by the presence of 1-pentanol. Rice flour (FR_SA) and buckwheat flour (FGS_SA), differ from the other samples by the presence of 2-propyl-1-heptanol, a compound that was also found in the PREP_bread_SH bread mix. This difference is important to confirm the use of this type of flour in the preparations. It is possible to assume that the alcohol originates from the presence of at least one of the two ingredients. This initial assumption was confirmed by the list of ingredients in the bread-making mixture mentioned earlier. 2-hexyl-1-octanol is present only in pastry mix, but not in packaged pastry.

Considering some of the foods having flour as an ingredient, it is noted that only in the bread samples PS_SH and PI_CO and in a cracker sample (CR_NU), 1-hexanol, 1-pentanol and 2-octen-1-ol, alcohols already were identified in flours. In all other processed foods, the analytes of the flours are not found. This may be due to the loss of these compounds during processing and baking. Among these, 2-octen-1-ol is almost always present in flours but absent in final products. Four of the analysed samples (PG_NU, CR_CE, rosemary, and vanillin) do not contain alcohols in their volatile profile (Fig. 2B and Fig. 1 in supplementary material).

In the case of the breadcrumb (PG_NU) and cracker (CR_CE) samples, given the presence of yeast in the ingredient list, the absence of alcohols could be explained by high temperature in the product processing steps and the volatility of the analytes. Fig. 2B also shows that 1,3-butanediol is present only in the BG_GU, FG_GA, BG_CO and FCG_CO cookie samples, all containing cocoa and/or chocolate chips, which are assumed to be responsible for the presence of this alcohol. This hypothesis is confirmed by the presence of 1,3-butanediol in the cocoa sample (Fig. 1 in supplementary material) and the absence of this compound in the plain shortbread (B_GU). In the above four cookie samples, another common compound is 2,3-butanediol, which could result from fermentation reactions or the Maillard reaction.

By comparing the results of the analysis of the alcohols of the four bread samples, two of white bread (PB_CE and PB_NU) and two of rustic bread with wholemeal and/or seed flours (PI_CO and PS_SH), it can be seen that the first two profiles of the volatile components are quite similar and consist mainly of 2-pentanol and 2-furanmethanol (Furfuryl alcohol), while the other two are different both from each other and from those of the white bread samples. The 2-Furanmethanol (Furfuryl alcohol) is present in 9 of the analysed samples (bread, breadsticks, and several sweet snacks) and is absent only in the PS_SH sample. This compound is present at the highest concentration in the PB_NU white bread sample, with a concentration of 0.18 mg/Kg. In the GR_SC breadsticks and B_GU plain shortbread samples, although present in lower concentrations than that of the PB_NU sample, it is the only alcohol. The 2-Furanmethanol could have different origins: it is produced during the caramelization process of sugars or by the Maillard reaction (Pico, Khomenko, Capozzi, Navarini, & Biasioli, 2020). According to other authors, 2-furanmethanol could be the product of furfural reduction by the action of *Saccharomyces cerevisiae* (Pico, Martinez, et al., 2017).

The 1,2-propanediol was identified only in 5 samples, all sweet snacks. This compound is used in baked products as carrier/solvent to dissolve the antioxidant additives (Giuliano & Stein, 1975). This compound is present in samples at concentrations ranging from 0.163 mg/Kg (PLUM_SO) to 1.2 mg/Kg (FCG_CO).

The 2-decen-1-ol is only present in the pasta sample (PASTA_SE) and paprika.

The 1,2-cyclopentanediol, 3-phenyl-2-butanol and 2,4-undecadien-1-ol are present at low concentrations only in the crackers sample (GALLETTE_SC).

### 3.2 Aldehydes and ketones

**Fig. 3:**
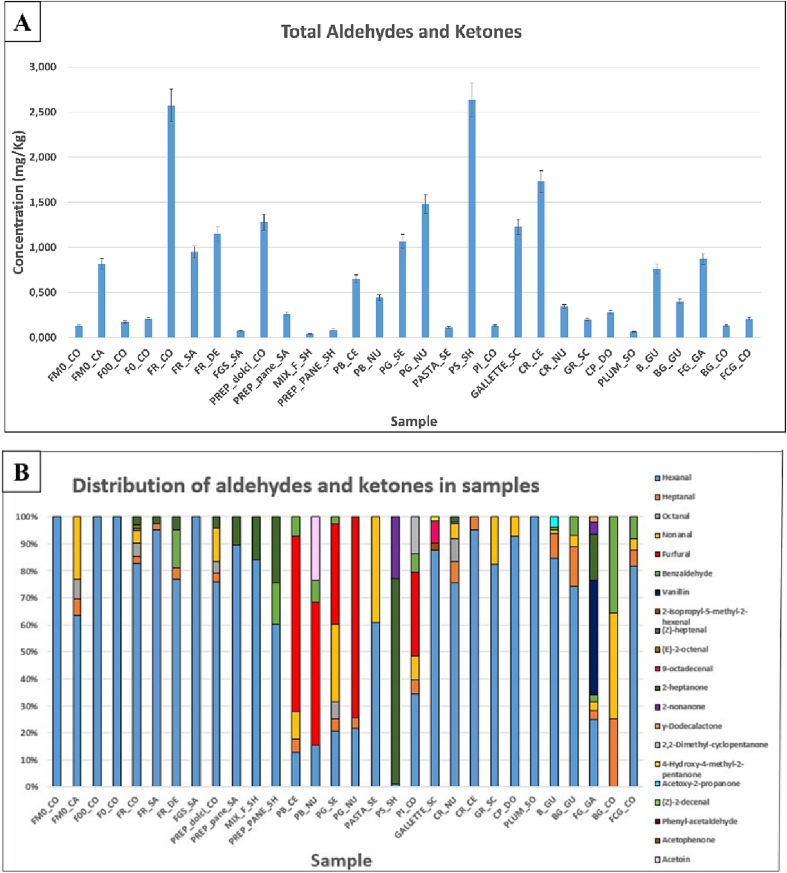
Total concentration (mg/Kg) of aldehydes and ketones in samples (A), percentage distributions of aldehydes and ketones in samples (B).

The concentrations of aldehydes and ketones were calculated using 2-nonanone as standard. In Fig. 3A the total concentrations are reported. It ranged from 0.040 mg/Kg (MIX_F_SH) to 2.6 mg/Kg (FR_CO).

Among the aldehydes, hexanal is the most frequent (Fig. 3A), in fact, it was identified in all samples except cookies (BG_CO) with concentrations ranging from 0.029 mg/Kg (PS_SH) to 2.1 mg/Kg (FR_CO). In decreasing order of concentration, it is heptanal (in 16 samples and cocoa), followed by nonanal (in 15 samples and cocoa) and octanal (in 5 samples).

In general, in foods, aldehydes are considered marker compounds of oxidation processes. Lipid oxidation is one of the main reactions involved in food spoilage and can occur in the production stages and during storage; this reaction can contribute to flavor changes. The oxidation of lipids occurs mainly in foods rich in fats but can also occur in flours, products typically poorer in fatty acids, as was observed in this study (Azarbad & Jelen, 2014). In fact, the presence of volatile compounds deriving from oxidative processes, in addition to being correlated to the quantity and nature of the lipids contained in the raw materials, is also correlated to the processing temperature of the food. Indeed, the enzymatic activity decreases with increasing temperature. Lipoxygenase, the enzyme that catalyses the no reversible oxidation reaction that leads from fatty acids to the formation of carbonyl compounds, is denatured as early as 50 °C (Quarantelli, Righi, Renzi, & Bonomi, 2003). In this regard, hexanal concentrations has significantly lower in bread samples, a product that typically has high cooking temperatures (> 200 °C) than the other foods.

However, the mechanisms of reaction and the rate of oxidation depend on many other factors, such as the presence of antioxidants, pH, and storage conditions (Mozuraityte, Kristinova, & Rustad, 2016); in fact, it is known that the oxidation process is accelerated by factors such as light, temperature, oxygen availability and humidity (Pastorelli, Valzacchi, Rodriguez, & Simoneau, 2006).

Hexanal is a typical compound derived from the oxidation of linoleic and arachidonic acids; its presence in foods is often used as a marker for assessing lipid oxidation. Bredie and other researchers (Bredie, Mottram, & Guy, 2002) have shown that it is the main aldehyde formed in wheat flour during one of the processing steps.

Heptanal, octanal, nonanal and the 2-decenal can be formed by fatty acid auto-oxidation processes (Maire et al., 2013). In addition to the degradation of unsaturated fatty acids, such as oleic, linoleic, and linolenic acids, aldehydes can also result from Strecker degradation (Chai et al., 2019).

The 4-hydroxy-3-methoxybenzaldehyde (vanillin), is only present in chocolate chip shortbread (FG_GA) with a concentration of 0.37 mg/Kg, probably prepared by adding commercial vanillin. The latter was also analysed and appears to be quite pure (Fig. 2 in supplementary material). In addition to being a common flavor in pastry production, vanillin is also a food antioxidant agent. (Burri, Graf, Lambelet, & Loliger, 1989).

Benzaldehyde is present only in 10 samples and in cocoa with concentrations ranging from 0.008 mg/Kg (B_GU) to 0.68 mg/Kg (Cacao).

The 2-Heptanone is present in 10 samples and particularly in all rice flours (FR_CO, FR_SA, FR_DE) and in three end products: PS_SH, CR_NU and FG_GA.

The 2-Furancarbaldehyde (Furfural), which may result from the Maillard reaction or from caramelization processes of sugars (Pico ed. al., 2020), is present in 3 bread samples (PB_CE, PB_NU and PI_CO) and in breadcrumb samples (PG_NU and PG_SE). The concentration of the above analytes varied between 0.041 mg/Kg (PI_CO) to 1.1 mg/Kg (PG_NU). Acetoin (3-Hydroxy-2-butanone) is only present in the white bread sample (PB_NU) with a concentration of 0.10 mg/Kg. Acetoin is mainly formed in the glycolysis of pyruvic acid in yeast-induced fermentation, but it could also be produced by the Maillard reaction during baking (Pico, Bernal, & Gòmez, 2017).

2-Nonanone is only present in two samples, chia seed bread PS_SH and chocolate chip shortbread FG_GA, respectively, at concentrations of 0.60 mg/Kg and 0.041 mg/Kg.

The FR_CO rice flour sample also contains (*Z*)-heptenal and (E)-2-octenal, with concentrations of 0.023 mg/Kg and 0.029 mg/Kg, respectively. The (E)-2-octenal originates by the auto-oxidation of linoleic acid (Maire et al., 2013).

Cocoa also contains other analytes (Fig. 2 in supplementary material), including aldehydes and ketones, besides those already mentioned, including phenylacetaldehyde (0.40 mg/Kg), acetophenone (0.034 mg/Kg) and (Z)-2-decenal (0.13 mg/Kg). While as mentioned before, (Z)-2-decenal may result from lipid oxidative processes, phenylacetaldehyde from the metabolism of some microorganisms. This hypothesis is in good agreement with Birch et al. (Birch, Petersen, & Hansen, 2013), which showed that the presence of phenylacetaldehyde is positively affected by yeast concentration. Phenylacetaldehyde, characterized by its odour reminiscent of honey, makes an important sensory contribution to the volatile profile of the food in which it is found (Pico, Martinez, et al., 2017). Finally, some ketones were identified in one sample, γ-dodecalactone in the FG_GA sample (0.016 mg/Kg), 4-hydroxy-4-methyl-2-pentanone in the GALLETTE_SC (0.020 mg/Kg), and 2,2-dimethyl-cyclopentanone in the PI_CO (0.018 mg/Kg), therefore, they were not considered significant for the purpose of this research.

### 3.3 Heterocyclic compounds

**Fig. 4:**
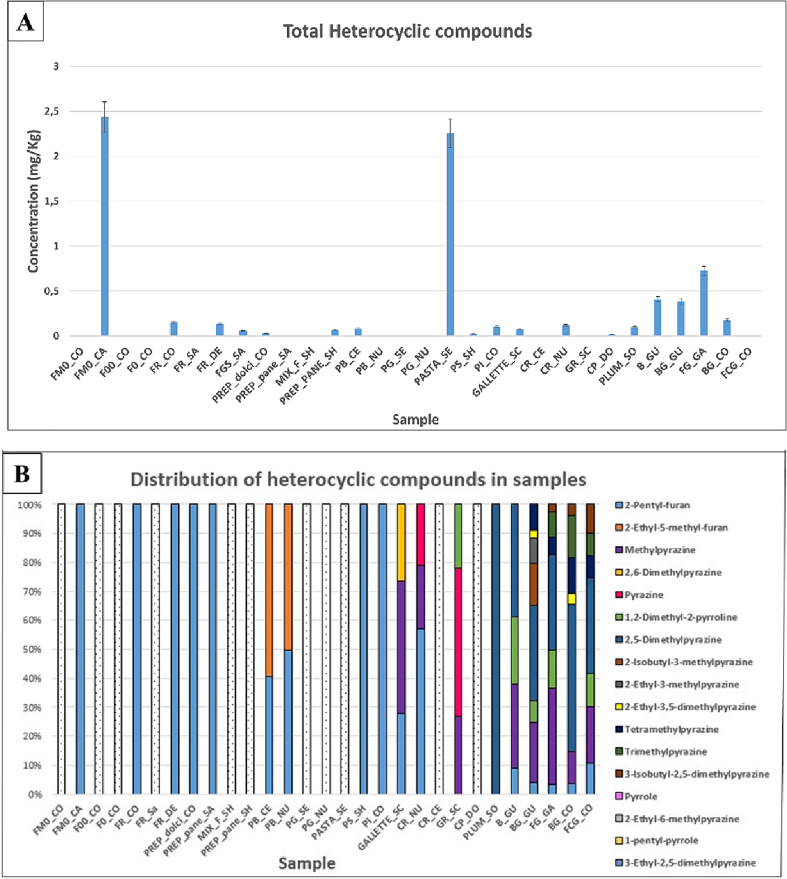
Total concentration (mg/Kg) of heterocyclic compounds in the samples (A), percentage distributions of heterocyclic compounds in samples (B).

Using the pyrazine calibration line, the concentrations of the heterocyclic compounds were calculated. As can be seen from Fig. 4A, the total concentrations in the food samples, without considering added flavorings, are between the limit of quantification and 2.4 mg/Kg (FM0_CA).

In general, heterocyclic compounds are less present in the analysed foods, as can be seen in Fig. 4B compared to the other classes of analytes already described.

The 2-Pentyl-furan is the most frequent heterocyclic compound, and it is present in 17 samples, in 7 of which it represents the only one of the heterocyclic compounds. It is present in flours and final products, with concentrations ranging from 0.009 mg/Kg (B_GU) to 2.4 mg /Kg (FM0_CA).

The 2-Pentyl-furan can be produced by various reactions, such as during some fermentation processes by Lactobacillus or during cooking by *trans*-2-nonenal, a lipid oxidation product of linoleic acid (De Luca et al., 2021). In the white bread samples (PB_CE and PB_NU), pentyl-furan accounts for 41% and 50% of the total heterocyclic compounds, respectively; the rest of the heterocyclic compounds in these two samples are 2-ethyl-5-methyl-furan.

Furans can be originated by thermal degradation and/or rearrangement of carbohydrates during the Maillard reaction (Cirlini, & Dall'Asta, C., Silvannini, A., Beghè, D., Fabbri, A., Galaverna, G., & Ganino, T., 2012). As for pyrazines, these are mainly present in sweet snacks and some salty ones. One type of alkylpyrazine has been detected in cookie samples, those containing cocoa and/or chocolate. Indeed, these analytes have been considered an important class of compounds that contribute to the aroma of many foods, especially those subjected to roasting, including cocoa (Keeney, 1972). Indeed, heat treatment induces the Maillard reaction, which can lead to the formation of heterocyclic nitrogen compounds, including alkyl pyrazines, but also pyrrole derivatives (Starowicz & Zielinski, 2019).

Methylpyrazine was present in all cookie samples (B_GU, BG_GU, BG_CO, FG_GA, FCG_CO) and in 3 analysed salty snacks (GALLETTE_SC, CR_NU and GR_SC) in concentrations ranging from 0.017 mg/Kg (CR_NU) to 0.13 mg/Kg (FG_GA).

This compound was also found in the cocoa sample at a concentration of 3.6 mg/Kg (Fig. 3 in supplementary material).

Tetramethylpyrazine is also present in all cookies containing cocoa and/or chocolate (BG_GU, FG_GA, BG_C AND FCG_ CO) and is also present in cocoa sample at a concentration of 3.5 mg/Kg. This could confirm the correlation between the presence of pyrazines and that of this ingredient.

The 2-ethyl-3,5-dimethylpyrazine, 2-ethyl-3-methylpyrazine and 2-isobutyl-3-methylpyrazine are also present in the volatile compound profile of the BG_GU chocolate chip cookie sample. Pyrrole, 1-pentyl-pyrrole, 2-ethyl-6-methylpyrazine and 3-ethyl-2,5-dimethylpyrazine are also present in the cocoa sample. These differences may be associated with the unlike composition of the raw material from which the cookies were prepared and that the analysed cocoa is the commercial one.

The 2,5-Dimethylpyrazine is present in all the sweet snacks, at concentrations ranging from 0.017 mg/kg (PLUM_SO) to 0.37 mg/Kg (BG_CO) and in the cocoa sample, where it is present at a significantly higher concentration (6.1 mg/Kg).

Trimethylpyrazine and 3-isobutyl-2,5-dimethylpyrazine were identified in only 3 cookie samples (BG_GU, BG_CO, FG_GA), and is not present in cocoa.

The 2,6-Dimethylpyrazine was identified only in the GALLETTE_SC sample.

### 3.4 Terpenes

**Fig. 5:**
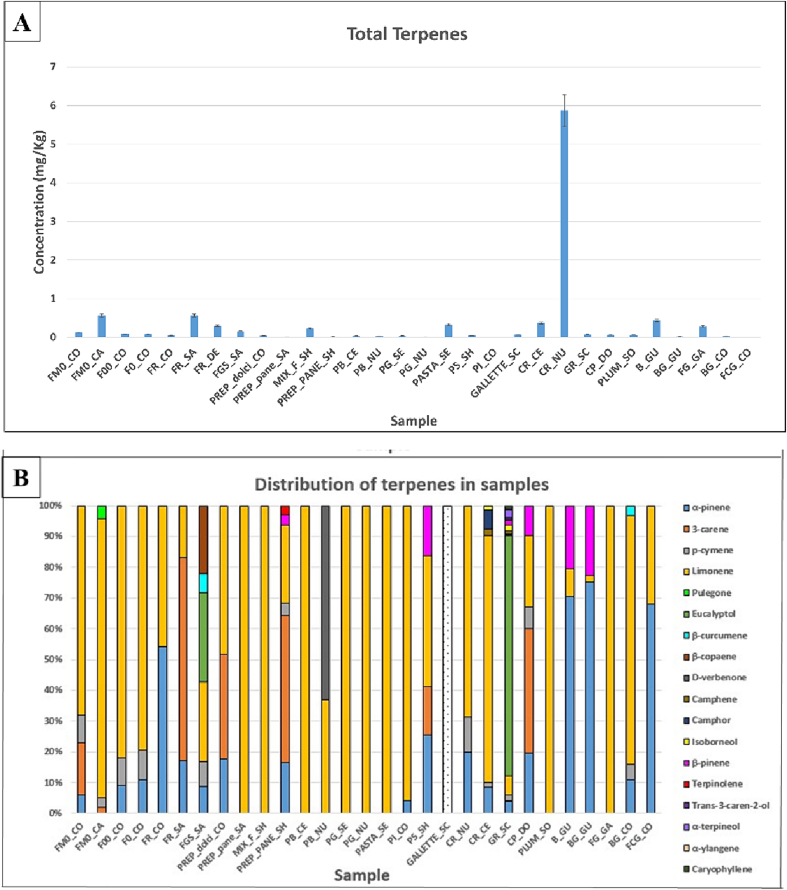
Total concentration (mg/Kg) of terpenes in samples (A), percentage distributions of terpenes in the samples (B).

Another class of compounds identified in the gluten-free samples is terpenes, which includes monoterpenes, sesquiterpenes. Using the camphor as standard, the concentrations of terpenes in the samples were calculated. As can be seen from Fig. 5A, without considering samples 31–34, the total concentrations ranged from 0.007 mg/Kg (PASTA_SE) to 5.9 mg/Kg (GR_SC).

The identification of these analytes in the analysed foods can be traced to a natural origin. In fact, terpenes are known to be secondary metabolites produced by different plants. Terpenic compounds identification can be used to discriminate the territory of origin of a specific ingredient (De Flaviis et al., 2021). In addition, some terpenes such as α-pinene, limonene, and β-pinene are used as food additives; these, in fact, represent aroma precursors that can produce, through bioconversion processes, VOCs that improve food aroma (Vespermann et al., 2017).

Limonene is presents in all but one sample (GALLETTE_SC) with concentrations ranging from 0.005 mg/Kg (MIX_F_SH) to 0.51 mg/Kg (FM0_CA). *α*-pinene is present in 18 samples with concentrations ranging from 0.008 mg/Kg (FM0_CO and F0_CO) to 0.23 mg/Kg (GR_SC). In contrast, *β*-pinene is only present in 6 samples, with concentrations ranging from 0.007 mg/Kg (PREP_pane_SH and CP_DO) to 0.098 mg/Kg (BG_GU). Pulegone is only present in a Manitoba 0 wheat flour (FM0_CA) with a concentration of 0.023 mg/Kg. The 3-Carene is present in 7 samples with concentrations ranging from 0.007 mg/Kg (PS_SH) to 0.37 mg/Kg (FR_SA). D-Verbenone is present in two samples, white bread sample (PB_NU) with a concentration of 0.019 mg/Kg and in a rosemary breadsticks sample (GR_SC) with a concentration of 0.022 mg/Kg. This compound could be produced by bioconversion processes from terpenes such as *α*-pinene and *β*-pinene (Agrawal & Joseph, 2000).

Camphor was only identified in rosemary cracker sample (CR_CE) with a concentration of 0.023 mg/Kg. Camphene is also present in the same sample with a concentration of 0.008 mg/Kg, a compound also identified in GR_SC sample with a higher concentration (0.049 mg/Kg). p-cymene is present in 11 samples with concentrations ranging from 0.005 mg/Kg (CP_DO) to 0.100 mg/Kg (GR_SC). *β*-Copaene is only present in the buckwheat flour sample (FGS_SA) with concentration 0.065 mg/Kg. Isoborneol is present in 2 samples, CR_CE and GR_SC, with respective concentrations of 0.005 mg/Kg and 0.11 mg/Kg. *β*-Curcumene is present in 3 samples (FGS_SA, GR_SC, BG_CO) with concentrations ranging from 0.009 mg/Kg to 0.018 mg/Kg. Eucalyptol was only found in the buckwheat flour sample (FGS_SA) and rosemary breadsticks (GR_SC), in amounts of 0.085 mg/Kg and 4.58 mg/Kg, respectively.

Terpinolene is present in 2 samples: in the bread mix (PREP_bread_SH) where it is present at concentration of 0.007 mg/Kg and in the rosemary breadsticks sample (GR_SC) where it is present in an amount of 0.029 mg/Kg.

The rosemary breadsticks sample is the richest in terpenes, in fact, in addition to those already mentioned, the following compounds *trans*-3-caren-2-ol (0.033 mg/Kg), *α*-terpineol (0.15 mg/Kg), *α*-ylangene (0.018 mg/Kg) and caryophyllene (0.057 mg/Kg) are also present. The abundance and multiplicity of terpenes in this sample can be traced to the presence of rosemary.

In fact, analysing rosemary flavor (Fig. 4 in supplementary material), as expected, this contains 18 compounds belonging to the terpene class, many of which correspond those identified in the breadstick samples (GR_SC).

Paprika, among the other flavorings considered, contains 9 terpenes, while only limonene is present in cocoa. Vanillin aroma has no terpenes in its volatile profile.

## 4 Conclusions

The developed analytical procedure represents a convenient approach for characterizing the volatile profile of foods. In fact, the HS-SPME/GC–MS analysis used is a simple, rapid, and semiautomatic method by which different types of samples can be analysed without the need for pretreatment and the use of solvents. This work constitutes an analytical approach for the quantification of VOCs in complex matrices such as foods. In this regard, to consider possible matrix effects, for the quantification of the analytes, all solid standards were prepared using corn starch. The optimization of the method allowed the characterization of the volatile component profiles of the gluten free food samples, including flours, sweet and savoury baked foods, and flavorings. In particular, the analytical method was used to quantify 127 compounds (alcohols, aldehydes, ketones, heterocyclic compounds, terpenes alkanes, benzene derivatives, esters, and other compounds) in 30 gluten free food samples.

From the results the most represented compounds are those derived from fermentation processes, Maillard reaction and lipid oxidation.

From the analysis of the data obtained, it is possible to conclude that the different volatile profiles identified, in good agreement with the literature data, allow the different samples to be discriminated and therefore represent almost a fingerprint. This evidence constitutes an important point in being able to monitor possible food counterfeiting and contamination, but not only that, in fact, the detection of alterations in the volatile profile of a food can provide information on its state of preservation and healthiness, especially, by monitoring target compounds of degradative processes.

Although the purpose of this work was not to assess the storage status of food, several compounds that can be used as markers of degradative processes, such as hexanal, were identified. In conclusion, therefore, we can say this study has expanded scientific knowledge related to this area of research by providing a valid methodology of analysis applicable to foods with and without gluten. In the future, aging studies could be carried out using the above method monitoring aldehyde concentrations, which could provide interesting information, also useful for the improvement of food packaging.

## CRediT authorship contribution statement

**Santino Orecchio:** Writing – review & editing, Writing – original draft, Validation, Resources, Methodology, Conceptualization. **Antonella Maggio:** Writing – review & editing, Writing – original draft, Validation, Supervision, Resources, Methodology, Funding acquisition, Conceptualization.

## Declaration of competing interest

None.

## Data Availability

Data will be made available on request.

## Appendix A Supplementary data

Percentage distributions of alcohols, aldehydes and ketones, heterocyclic compounds and terpenes in flavorings. Image 1

## References

[bb0005] Agrawal R., Joseph R. (2000). Bioconversion of alpha pinene to verbenone by resting cells of aspergillus Niger. Applied Microbiology and Biotechnology.

[bb0010] Azarbad M.H., Jelen H. (2014). Determination of hexanal- an Indicator of lipid oxidation by static headspace gas chromatography (SHS-GC) in fat-rich food matrices. Food Analytical Methods.

[bb0015] Birch A.N., Petersen M.A., Hansen A.S. (2013). The aroma profile of wheat bread crumb influenced by yeast concentration and fermentation temperature. LWT - Food Science and Technology.

[bb0020] Bredie W.L.P., Mottram D.S., Guy R.C.E. (2002). Effect of temperature and pH on the generation of flavor volatiles in extrusion cooking of wheat flour. Journal of Agricultural and Food Chemistry.

[bb0025] Burri J., Graf M., Lambelet P., Loliger J. (1989). Vanillin: More than a flavoring agent – A potent antioxidant. Journal of Science of Food and Agriculture.

[bb0030] Chai D., Li C., Zhang X., Yang J., Liu L., Xu X., Du M., Wang T., Chen Y., Dong L. (2019). Analysis of volatile compounds from wheat flour in the heating process. International Journal of Food Engineering.

[bb0035] Cirlini M., Dall'Asta C., Silvannini A., Beghè D., Fabbri A., Galaverna G., Ganino T. (2012). Volatile fingerprinting of chestnut flours from traditional Emilia Romagna (Italy) cultivars. Food Chemistry.

[bb0040] Cuadros-Rodríguez L., Bagur-González M., Sánchez-Viñas M., González-Casado A., Gómez-Sáez A. (2007). Principles of analytical calibration/quantification for the separation sciences. Journal of Chromatography. A.

[bb0045] De Flaviis R., Sacchetti G., Mastrocola D. (2021). Wheat classification to its origin by an implemented volatile organic compounds analysis. Food Chemistry.

[bb0050] De Luca L., Aiello A., Pizzolongo F., Blaiotta G., Aponte M., Romano R. (2021). Volatile organic compounds in breads prepared with different sourdoughs. *Applied Science*s.

[bb0060] Drakula S., Mustac N.C., Novotni D., Vouko B., Krpan M., Hruskar M., Curic D. (2021). Optimization and validation of a HS-SPME/GC-MS method for the analysis of gluten-free bread volatile flavor compounds. Food Analytical Methods.

[bb0065] Giuliano R., Stein M.L. (1975).

[bb0070] Hansen A., Hansen B. (1994). Influence of wheat flour type on the production of flavor compounds in wheat sourdough. Journal of Cereal Science.

[bb0075] Jelen H.H., Majcher M., Dziadas M. (2012). Microextraction techniques in the analysis of food flavor compounds: A review. Analytica Chimica Acta.

[bb0080] Keeney P.G. (1972). Various interactions in chocolate flavor. Journal of the American Oil Chemists Society.

[bb0085] Maggio A., Orecchio S. (2018). Fatty acid composition of gluten-free food (bakery products) for celiac people. Foods.

[bb0090] Maire M., Rega B., Cuvelier M.-E., Soto P., Giampaoli P. (2013). Lipid oxidation in baked products: Impact of formula and process on the generation of volatile compounds. Food Chemistry.

[bb0095] Makharia G.K., Singh P., Catassi C., Sanders D.S., Leffler D., Affendi R., Ali R., Bai J.C. (2022). The global burden of coeliac disease: Opportunities and challenges. Nature Reviews. Gastroenterology & Hepatology.

[bb0100] Mozuraityte R., Kristinova V., Rustad T. (2016). Oxidation of food components. Encyclopedia of Food and Health..

[bb0105] Nissen L., Bordoni A., Gianotti A. (2020). Shift of volatile organic compounds (VOCs) in gluten-free hemp-enriched Sourdoungh bread: A Metabolomic approach. Nutrients.

[bb0110] Orecchio S., Amorello D., Raso M., Barreca S., Lino C., Di Gaudio F. (2014). Determination of trace elements in gluten-free food for celiac people by ICP-MS. Microchemical Journal.

[bb0115] Orecchio S., Amorello D., Raso M., Barreca S., Lino C., Di Gaudio F. (2015). Determination of macro elements in gluten-free food for celiac people by ICP-OES. Life safety and security.

[bb0120] Pacynski M., Zawirska Wojtasiak R., Mildner-Szkudlarz S. (2015). Improving the aroma of gluten-free bread. LWT - Food Science and Technology.

[bb0125] Pastorelli S., Valzacchi S., Rodriguez A., Simoneau C. (2006). Solid phase microextraction method for the determination of hexanal in hazelnuts as an indicator of the interaction of active packaging materials with food aroma compounds. Food Additives and Contaminants.

[bb0130] Pico J., Martinez M.-M., Bernal J., Gòmez M. (2017). Impact of frozen storage time on the volatile profile of wheat bread crumb. Food Chemistry.

[bb0135] Pico J., Khomenko I., Capozzi V., Navarini L., Biasioli F. (2020). Real-time monitoring of volatile compounds losses in the oven during baking and toasting of gluten-free bread Dounghs: A PTR-MS evidence. Foods.

[bb0140] Pico J., Martinez M.M., Bernal J., Gomez M. (2017). Evolution of volatile compounds in gluten-free: From dough to crumb. Food Chemistry.

[bb0145] Quarantelli A., Righi F., Renzi M., Bonomi A. (2003). Processi ossidativi negli alimenti di origine vegetale. Ann. Fac. Medici. Vet. Di Parma..

[bb0150] Rajhi I., Baccouri B., Rajhi F., Mhadhbi H., Flamini G. (2021). Monitoring the volatile compound status of whole seeds and flours of legume cultivars. Food Bioscience.

[bb0155] Starowicz M., Zielinski H. (2019). How Maillard reaction influences sensorial properties (color, flavor and texture) of food products?. Food Reviews International.

[bb0160] Vespermann K.A., Paulino B.N., Barcelos M.C., Pessôa M.G., Pastore G.M., Molina G. (2017). Biotransformation of α- and β-pinene into flavor compounds. Applied Microbiology and Biotechnology.

