# Supplementary material for: Matrix-matched quantification of volatile organic compounds (VOCs) in gluten free flours and bakery products
Source: Food Chem X. 2024 May 21;22:101399. doi: 10.1016/j.fochx.2024.101399 (PMC11152647; doi:10.1016/j.fochx.2024.101399)
Supplement: Supplementary file 1 — Percentage distributions of alcohols, aldehydes and ketones, heterocyclic compounds and terpenes in flavorings. [file mmc1.docx]

Supplementary material

Matrix-matched quantification of volatile organic compounds (VOCs) in gluten free food

Antonella Porrello, Santino Orecchio, Antonella Maggio*

*Department of Biological, Chemical and Pharmaceutical Sciences and Technologies (STEBICEF), University of Palermo, Viale delle Scienze, Palermo, building 17, Italy.*

* Corresponding author*: Antonella Maggio*

E-mail: [antonella.maggio@unipa.it](mailto:antonella.maggio@unipa.it)

[antonella.porrello@unipa.it](mailto:antonella.porrello@unipa.it)

[santino.orecchio@unipa.it](mailto:santino.orecchio@unipa.it)

[antonella.maggio@unipa.it](mailto:antonella.maggio@unipa.it)

**Abstract**

This study deals with characterization of the profiles of VOC of gluten-free foods to highlight the possible presence of compounds responsible for organoleptic alterations, precursors of flavors, contaminants, and key odours, evaluating the potential of fingerprinting to differentiate raw material.

For the quantification, the matrix matched method was used with corn starch solid standards. It was applied to consider possible matrix effects allowing for an appropriate analysis. In this work, the analysis of the compounds that make up the aroma of a gluten-free food has been characterized by two types of approaches: the first by the determination of VOCs to know the profile of the volatile components of the food; the second represented by the analysis of compounds important for assessing the quality of the food, the production processes, and the storage conditions.

**Keywords**

Celiac disease; gluten-free flour; gluten-free bakery products; VOCs; GC-MS.


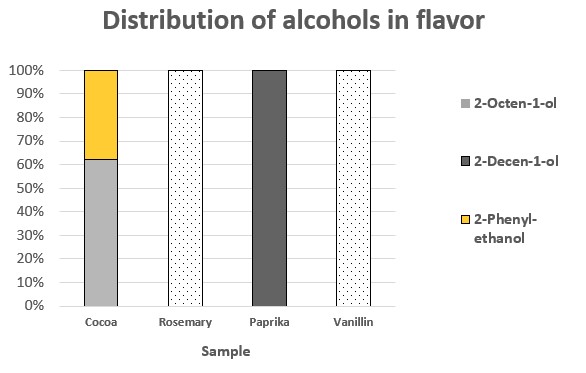


Fig.1 Percentage distributions of alcohols in flavorings.


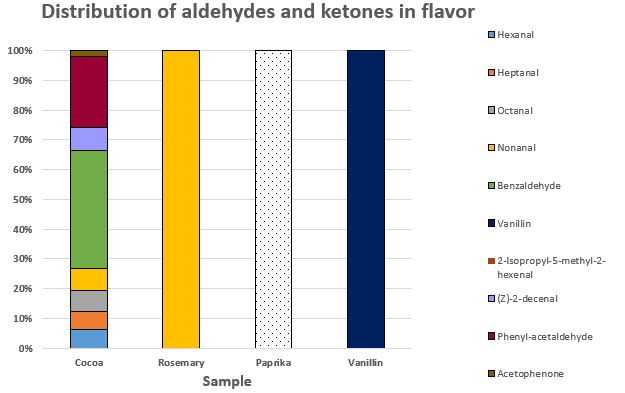


Fig.2 Percentage distributions of aldehydes and ketones in flavorings.


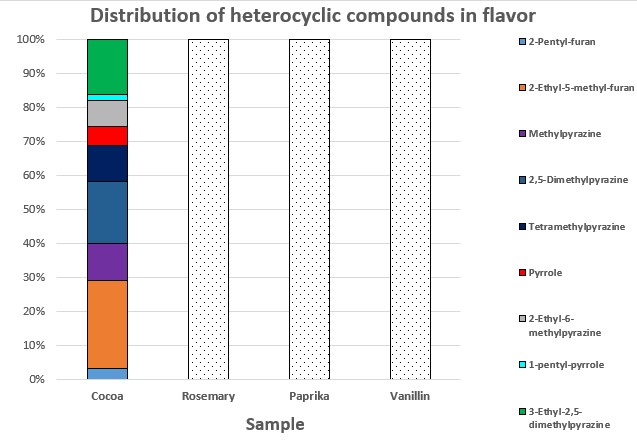


Fig.3 Percentage distributions of heterocyclic compounds in flavorings.


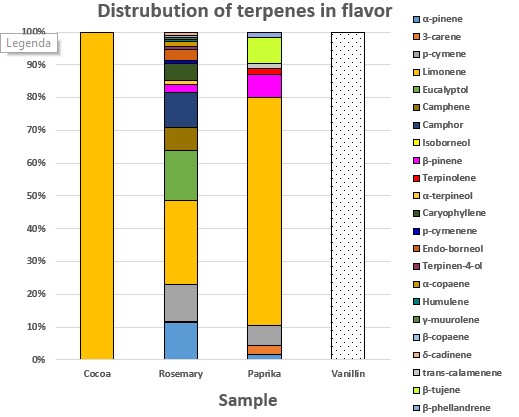


Fig.4 Percentage distributions of terpenes in flavorings.
